# Electronic case report forms generation from pathology reports by ARGO, automatic record generator for onco-hematology

**DOI:** 10.1038/s41598-021-03204-z

**Published:** 2021-12-10

**Authors:** Gian Maria Zaccaria, Vito Colella, Simona Colucci, Felice Clemente, Fabio Pavone, Maria Carmela Vegliante, Flavia Esposito, Giuseppina Opinto, Anna Scattone, Giacomo Loseto, Carla Minoia, Bernardo Rossini, Angela Maria Quinto, Vito Angiulli, Luigi Alfredo Grieco, Angelo Fama, Simone Ferrero, Riccardo Moia, Alice Di Rocco, Francesca Maria Quaglia, Valentina Tabanelli, Attilio Guarini, Sabino Ciavarella

**Affiliations:** 1 Hematology and Cell Therapy Unit, IRCCS Istituto Tumori ‘Giovanni Paolo II’, Viale Orazio Flacco, 65, Bari, Italy; 2 Department of Electrical and Information Engineering, Politecnico of Bari, Bari, Italy; 3 Department of Mathematics, University of Bari Aldo Moro, Bari, Italy; 4 Pathology Department, IRCCS Istituto Tumori ‘Giovanni Paolo II’, Bari, Italy; 5 Clinical Engineering Unit, IRCCS Istituto Tumori ‘Giovanni Paolo II’, Bari, Italy; 6 Hematology, Azienda USL - IRCCS Di Reggio Emilia, Reggio Emilia, Italy; 7 Division of Hematology 1, AOU “Città Della Salute e Della Scienza di Torino”, Torino, Italy; 8 Department of Molecular Biotechnologies and Health Sciences, University of Torino, Torino, Italy; 9 Division of Hematology, Azienda Ospedaliero-Universitaria Maggiore Della Carità Di Novara, Novara, Italy; 10 Unit of Hematology, Azienda Ospedaliero-Universitaria Policlinico Umberto I, Roma, Italy; 11 Department of Medicine, Section of Hematology, University of Verona, Verona, Italy; 12 Division of Diagnostic Haematopathology, European Institute of Oncology, IRCCS, Milano, Italy

**Keywords:** Translational research, B-cell lymphoma, Data acquisition, Data integration, Pathology, Diagnostic markers, B-cell lymphoma

## Abstract

The unstructured nature of Real-World (RW) data from onco-hematological patients and the scarce accessibility to integrated systems restrain the use of RW information for research purposes. Natural Language Processing (NLP) might help in transposing unstructured reports into standardized electronic health records. We exploited NLP to develop an automated tool, named ARGO (Automatic Record Generator for Onco-hematology) to recognize information from pathology reports and populate electronic case report forms (eCRFs) pre-implemented by REDCap. ARGO was applied to hemo-lymphopathology reports of diffuse large B-cell, follicular, and mantle cell lymphomas, and assessed for accuracy (A), precision (P), recall (R) and F1-score (F) on internal (n = 239) and external (n = 93) report series. 326 (98.2%) reports were converted into corresponding eCRFs. Overall, ARGO showed high performance in capturing (1) identification report number (all metrics > 90%), (2) biopsy date (all metrics > 90% in both series), (3) specimen type (86.6% and 91.4% of A, 98.5% and 100.0% of P, 92.5% and 95.5% of F, and 87.2% and 91.4% of R for internal and external series, respectively), (4) diagnosis (100% of P with A, R and F of 90% in both series). We developed and validated a generalizable tool that generates structured eCRFs from real-life pathology reports.

## Introduction

Over the last few years, the complexity of clinical and biological data for a proper diagnosis and prognostication of onco-hematological diseases has remarkably increased, especially in the field of lymphomas^1,2^. In parallel, novel therapeutics found continue approvals from large, controlled trials, but missed parallel validation in the Real-World (RW) settings^3^. This major controversy claims for an urgent improvement of the capability to collect and share RW data with the final goal to support clinical and translational research^4^. Frequently, RW data are derived from fragmented sources as medical registries, electronic records, computerized patient order entries, individual databases, paper notes, as well as monocentric bio-banking-related annotations. Moreover, the common dearth of specialized data-entry professionals and the uneasy accessibility to data-extraction systems for most physicians accentuate the need for tools that facilitate the process of health data recording ^5^.

Natural Language Processing (NLP) is a consolidated technique to extract essential unstructured data from text, e.g. from diagnostic and prognostic notes^6–10^, widely adopted also in onco-hematology^11–16^. REDCap (Research Electronic Data CAPture) is a recognized platform of electronic case report forms (eCRFs) enabling rapid, high-quality and standardized annotation of data^17,18^. A potential bridge between NLP and eCRFs population is interposed by Optical Character Recognition (OCR), namely a state-of-the-art technology able to convert paper-based reports into digital formats to be further structured—possibly through NLP techniques—in electronic health records (EHR). Thus, OCR overcomes the need of integration between textual reporting and digital storage systems^19,20^.

Here, we describe the development of a NLP-based tool, named ARGO (Automatic Record Generator for Onco-hematology), to automatically convert RW reports in standardized eCRFs for data collection. To test its generalizability, we applied ARGO to a multicentric set of RW pathology reports of Non-Hodgkin Lymphomas (NHL) and validated its functionality, performance, and suitability for future translation into the daily practice.

## Results

### Electronic data collection workflow

**Figure 1:**
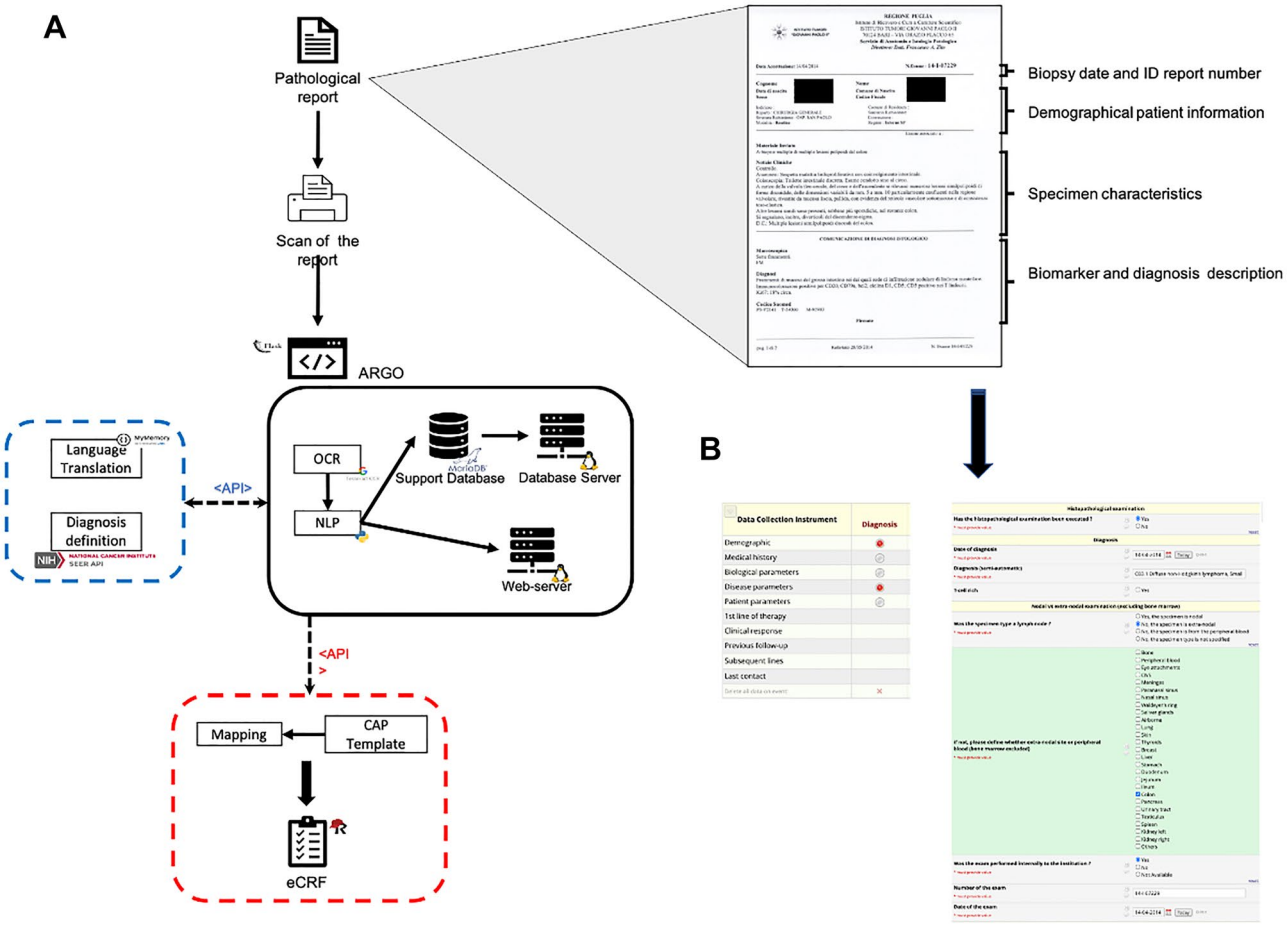
Graphical description of the framework. (**A**) Each paper-based report is manually transformed into an image file by a common digital scanner (right upside, an example of paper-based report from the Pathology Unit of the IRCCS Istituto Tumori “Giovanni Paolo II” of Bari, Italy). Then, the image is uploaded into ARGO through a web interface (black block), transformed in structured text through OCR and saved (by an NLP approach) as structured data in a database via webserver. “Diagnosis” attribution is carried out via API connecting ARGO with SEER servers (blue block). Finally, ARGO automatically populates eCRFs via API (red block). (**B**) Representative picture of REDCap dashboard for a single case report including “Demography” and “Disease parameters” forms (red bullets). Abbreviations. ARGO: Automatic Record Generator for Onco-hematology, OCR: Optical Character Recognition, NLP: Natural Language Processing, SEER: Surveillance, Epidemiology, and End Results, eCRFs: electronic Case Report Forms, API: Application Programming Interface, REDCap: Research Electronic Data-Capture, ID: Identification.

The capacity of ARGO to effectively automize eCRF generation was tested on both internal and external cohorts of NHL paper-based pathology reports, including Diffuse Large B-Cell Lymphoma (DLBCL), Follicular Lymphoma (FL), and Mantle Cell Lymphoma (MCL). ARGO read all the words in each template currently adopted at the Pathology Unit of the IRCCS Istituto Tumori “Giovanni Paolo II” as well as at Pathology Units of six additional Italian Hospitals. As illustrated in Fig. 1A, each histopathology report included several data organized in four main sections: (1) biopsy date and Identification (ID) report number; (2) patient demographical information; (3) specimen characteristics; and (4) biomarker and diagnosis description.

The first implementation step of ARGO pipeline consisted in the advantageous transformation of each paper-based report into an image file (.jpg extension) by using a common digital scanner. Thus, each report was uploaded on the ARGO application, which saved structured text into a support database, retrieved all the relevant data from the text, and transferred them directly into dedicated eCRFs. 233 out of 239 reports of the internal series and all those belonging to the external series (n = 93) were successfully converted in eCRF records. ARGO failed in converting six reports due to either low optical quality or their length (> 1 paper page). Figure 1B shows the main sections of each eCRF, which included both “demographic” and “disease” modules (see also Figure S1 from Supplementary Appendix), in a way consistent with the content of the corresponding original paper report. A video demonstrates ARGO’s functionality in Multimedia Appendix.

### Data retrieved from diagnostic reports

**Figure 2:**
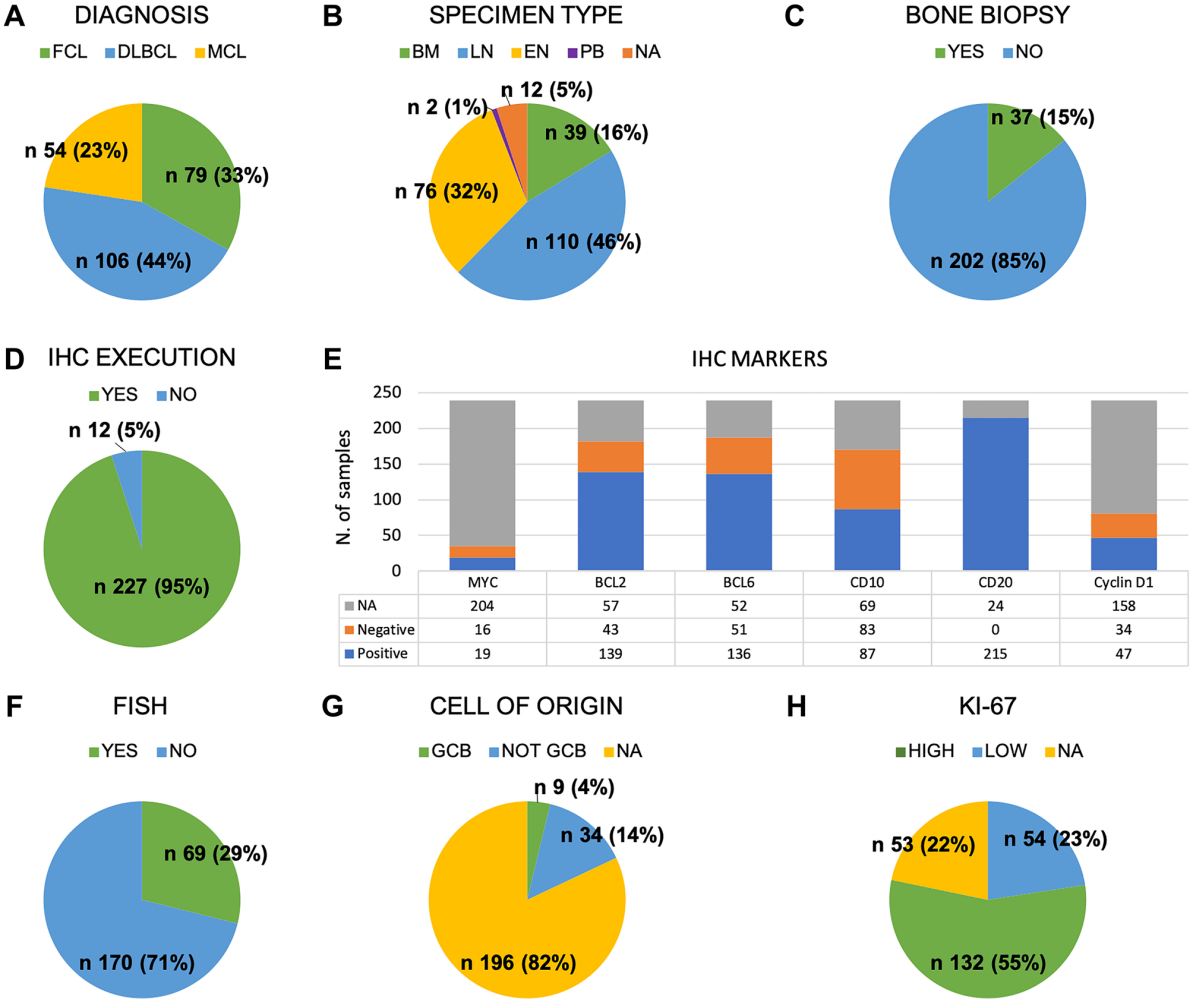
Characteristics of data retrieved from diagnostic reports. Graphical representation of diagnostic features, subdivided into specific fields, captured by ARGO from a total of n. 239 paper-based pathology reports of the internal series. Abbreviations. ARGO: Automatic Record Generator for Onco-hematology, FCL: Follicular Lymphoma, DLBCL: Diffuse Large B-cell Lymphoma, MCL: Mantle Cell Lymphoma, BM: Bone Marrow, LN: Lymph-Node, EN: Extra-Nodal, PB: Peripheral Blood, NA: Not Available, IHC: Immunohistochemistry, N: number, CD: Cluster of Differentiation, FISH: Fluorescent in situ hybridization, GCB: Germinal Center B-like.

Among the 239 paper-based reports retrieved from the internal series, 106, 79, and 54 were conclusive for a diagnosis of DLBCL, FL, and MCL, respectively (Fig. 2A). Overall, 110 diagnostic specimens were obtained from a tissue extracted from a lymph-node (LN), 76 were extra-nodal (EN), and 39 from bone marrow (BM), 2 from peripheral blood (PB), while for 12 cases this information was not available (Fig. 2B). In 85% of cases, a matched bone marrow biopsy was not available (Fig. 2C). Results from immunohistochemistry (IHC) staining for MYC, BCL2, BCL6, cluster of differentiation (CD)10, CD20, and Cyclin-D1 were available in 227 out of 239 cases and included a qualitative (positive/negative) assessment for the most relevant markers (Fig. 2D-E). A FISH (Fluorescent in situ hybridization) analysis (for MYC, BCL2, BCL6 or Cyclin-D1) appeared in the 29% of reports (Fig. 2F), whereas Cell of Origin (COO) categorization was reported in nearly 18% of cases (Fig. 2G). Of note, 186 out of 239 reports included the quantitative value of the Ki-67. Among these, 54 reported a value lower than 30% (Fig. 2H). Table 1 shows the full reports characteristics from the internal series.

**Table 1:** Characteristics of pathology reports.

	Internal series		External series	
	N	(%)	N	(%)
	239	(100.0)	93	(100.0)
**Diagnosis**				
DLBCL	106	(44.4)	49	(52.7)
FCL	79	(33.1)	24	(25.8)
MCL	54	(22.6)	20	(21.5)
**Specimen type**				
LN	110	(46.0)	53	(57.0)
EN	76	(31.8)	28	(30.1)
BM	39	(16.3)	12	(12.9)
PB	2	(0.8)	0	(0.0)
NA	12	(5.0)	0	(0.0)
**Bone biopsy**				
Yes	37	(15.5)	14	(15.1)
No	202	(84.5)	79	(84.9)
**IHC Execution**				
Yes	227	(95.0)	93	(100.0)
No	12	(5.0)	0	(0.0)
**IHC Markers**				
***MYC***				
Positive	19	(7.9)	30	(32.3)
Negative	16	(6.7)	8	(8.6)
NA	204	(85.4)	55	(59.1)
***BCL2***				
Positive	139	(58.2)	72	(77.4)
Negative	43	(18.0)	6	(6.5)
NA	57	(23.8)	15	(16.1)
***BCL6***				
Positive	136	(56.9)	59	(63.4)
Negative	51	(21.3)	11	(11.8)
NA	52	(21.8)	23	(24.7)
***CD10***				
Positive	87	(3.6)	40	(4.3)
Negative	83	(34.7)	39	(41.9)
NA	69	(28.9)	14	(15.1)
***CD20***				
Positive	215	(90.0)	84	(90.3)
Negative	0	(0.0)	2	(2.2)
NA	24	(10.0)	7	(7.5)
***Cyclin D1***				
Positive	47	(19.7)	21	(22.6)
Negative	34	(14.2)	22	(23.7)
NA	158	(66.1)	50	(53.8)
**FISH**				
Yes	69	(28.9)	5	(5.4)
No	170	(71.1)	88	(94.6)
**Cell of origin**				
GCB	9	(3.8)	14	(15.1)
Not GCB	34	(14.2)	19	(20.4)
NA	196	(82.0)	60	(64.5)
**Ki-67**				
< 30	54	(22.6)	17	(18.3)
≥ 30	132	(55.2)	58	(62.4)
NA	53	(22.2)	18	(19.4)

ARGO: Automatic Record Generator for Onco-hematology, FCL: Follicular Lymphoma, DLBCL: Diffuse Large B-cell Lymphoma, MCL: Mantle Cell Lymphoma, BM: Bone Marrow, LN: Lymph-Node, EN: Extra-Nodal, PB: Peripheral Blood, NA: Not Available, IHC: Immunohistochemistry, N: number, CD: Cluster of Differentiation, FISH: Fluorescent in situ hybridization, GCB: Germinal Center B-like.

Among the 93 paper-based reports retrieved externally from other six Italian centers, 49, 24, and 20 were conclusive for a diagnosis of DLBCL, FL, and MCL, respectively (Table 1). Overall, 53 diagnostic specimens were obtained from LN, 28 were EN, and 12 from BM. In 85% of cases, a matched bone marrow biopsy was not available. Results from IHC staining for *MYC, BCL2, BCL6, CD10, CD20,* and *Cyclin-D1* were available in 93 out of 93 cases and in external series included a qualitative (positive/negative) assessment for the most relevant biomarkers. A FISH analysis (for *MYC, BCL2, BCL6* or *Cyclin-D1*) appeared in the 5.4% of reports, whereas *COO* categorization was reported in nearly 35.5% of cases. Of note, 93 out of 93 reports included the quantitative value for *Ki-67*. Among these, 17 reported a value lower than 30%. Table 1 shows the full reports characteristics from the external series.

## Internal vs external validation

**Figure 3:**
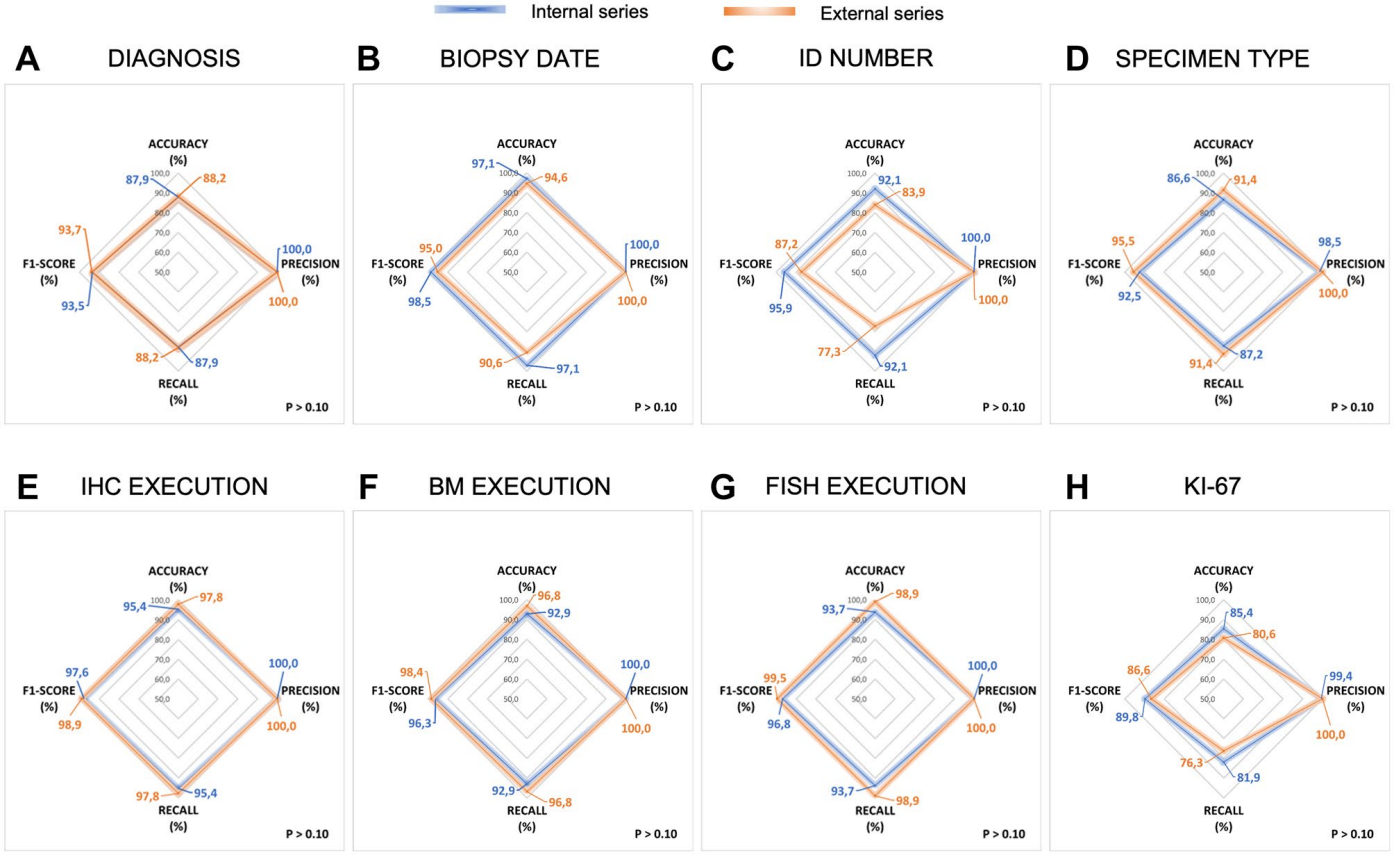
ARGO performance. Radar graphs indicate the performance metrics as percentage of accuracy, precision, recall and F1-score in different data fields for both internal and external series of pathology reports. Abbreviations. ID: Identification, IHC: Immunohistochemistry, BM: Bone Marrow, FISH: Fluorescent in situ hybridization.

Overall, ARGO detected 127,578 terms of interest and successfully generated EHR from 326 out of 332 processed histopathology reports. Figure 3 shows the comparative (internal vs. external series) post-hoc validation of ARGO performance for all the study data fields. For the “*DIAGNOSIS*” field, ARGO reached 88.9% vs. 87.9% (*p* > 0.05) of accuracy and recall, 93.5% and 93.7% of F1-score, also achieving 100% of precision in both series. For the “*BIOPSY DATE*” and the “*ID NUMBER*” fields of the internal series, all the applied metrics were > 90%. In comparison, accuracy, F1-score, and recall of external series for the “*BIOPSY DATE*” field were 94.6%, 95.0%, and 90.6%, respectively (*P* > 0.10), whereas “*ID NUMBER*” field ranged between 77.3% (recall from the external series) and 100.0% (precision from both internal and external series) (*P* > 0.10). For the “*SPECIMEN TYPE*” field, ARGO reached 86.6% vs. 91.4% of accuracy, 98.5% vs. 100.0% of precision, 92.5% vs. 95.5% of F1-score and 87.2% vs. 91.4% of recall (*P* > 0.10 in all instances). Similar high performance was observed for the “*IHC EXECUTION*” field (95.4% vs. 97.8% of accuracy and recall, 97.6% vs. 98.9% F1-score, and 100.0% of precision [*P* > 0.10]), although accuracy, recall and F1-score, but not precision (100.0%), slightly decreased as for single biomarker analyses (Supplementary Table S1). Similar results were also recorded the “*BM AND FISH EXECUTION*” fields. Finally, ARGO allowed the detection of “*Ki-67*”-related information with 85.4% vs. 80.6% (*P* > 0.10), 99.4% vs. 100.0% (*P* > 0.10), 81.9% vs. 76.3% (*P* > 0.10), and 89.8% vs. 86.6% (*P* > 0.10) of accuracy, precision, recall and F1-score, respectively. Overall, no significant differences between internal vs. external series were found in 14 out of 15 tested data fields (Supplementary Table S1). Of note, there is significant improvement (*P* < 0.01) in detecting the CD10 biomarker from the internal (67.4% of accuracy, 96.9% of precision, 55.9% of recall, and 70.9% of F1-score) compared to the external series (82.8% of accuracy, 98.5% of precision, 81.0% of recall, and 88.9% of F1-score).

To assess potential weaknesses of OCR in detecting data regarding single biomarkers, we selected 50 reports from the internal series with higher image resolution and reassessed the validation metrics (Table 2). Overall, recall and F1-score metrics improved of 12.9% and 9.1%, respectively. Moreover, we assessed the NLP performance on the internal series, independently of OCR. Interestingly, we observed an incremental trend of the recall for 7 of the 8 variables analyzed (“*DIAGNOSIS*” 87.9% vs. 90.0%; “*ID NUMBER*” 92.1% vs. 96.2%, “*SPECIMEN TYPE*” 87.2% vs. 92.7, “*IHC EXECUTION*” 95.4% vs. 95.8%, “*FISH EXECUTION*” 93.7% vs. 97.5%, “*BM EXECUTION*” 92.9% vs. 97.1%, and “*KI-67*” 81.9% vs. 89.4%). Only the field “*BIOPSY DATE*” showed a slight decrease from 97.1 to 96.2%, which we considered not relevant (Table 3).

**Table 2:** Comparison of ARGO performance in the whole vs. the top 50 reports^a^.

	PRECISION (P) = TP/(TP + FP)									RECALL (R) = TP/(TP + FN)									F1-SCORE = 2*(P*R)/(P + R)		
	All reports, N = 239				Top reports, N = 50^a^				Diff	All reports, N = 239				Top reports, N = 50^a^				Diff	All reports, N =239	Top reports N = 50^a^	Diff
DATA-FIELD	TP, N	FP, N	TP + FP	P, %	TP, N	FP, N	TP + FP, N	P, %	%	TP, N	FN, N	TP + FN, N	R, %	TP, N	FN, N	TP + FN, N	R, %	%	F1, %	F1, %	%
*MYC*	20	0	20	100.0	13	0	13	100.0	0.0	20	15	35	57.1	13	4	17	69.2	12.1	72.7	81.8	9.1
*BCL2*	130	2	132	98.5	28	0	28	100.0	1.5	130	55	185	71.4	28	5	33	84.8	13.4	82.2	91.8	9.6
*BCL6*	115	1	116	99.1	27	0	27	100.0	0.9	115	51	166	61.5	27	5	32	84.4	22.9	75.9	91.5	15.6
*CD10*	95	3	98	96.9	25	0	25	100.0	3.1	95	75	170	55.9	25	7	32	78.1	22.2	70.9	87.7	16.8
*CD20*	164	1	165	99.4	36	0	36	100.0	0.6	164	51	215	76.3	36	3	39	92.3	16.0	86.3	96.9	10.6
*Cyclin D1*	58	0	58	100.0	5	0	5	100.0	0.0	58	23	81	71.6	5	3	8	62.5	-9.1	83.5	76.3	-7.2
–	Mean (std)								1.0 (1.2)	Mean (std)								12.9 (11.7)	Mean (std)		9.1 (8.6)

ARGO: Automatic Record Generator for Onco-hematology, TP: True Positive, FP: False Positive, FN: False Negative, CD: Cluster of Differentiation, Diff: difference, std: standard deviation.

^a^Top 50 reports (internal series) with the highest optical resolution.

**Table 3:** Comparison of ARGO performance using OCR + NLP and NLP alone (internal series).

DATA-FIELD	PRECISION (%)			RECALL (%)			F1-SCORE (%)		
	OCR + NLP	NLP	Diff	OCR + NLP	NLP	Diff	OCR + NLP	NLP	Diff
*DIAGNOSIS*	100.0	100.0	0.0	87.9	90.0	2.1	93.5	94.7	1.2
*BIOPSY DATE*	100.0	100.0	0.0	97.1	96.2	– 0.8	98.5	98.1	– 0.4
*EXAM NUMBER*	100.0	100.0	0.0	92.1	96.2	4.2	95.9	96.2	0.3
*SPECIMEN TYPE*	98.5	99.5	1.0	87.2	92.7	5.5	92.5	96.0	3.5
*IHC EXECUTION*	100.0	100.0	0.0	95.4	95.8	0.4	97.6	95.8	– 1.8
*FISH EXECUTION*	100.0	100.0	0.0	93.7	97.5	3.8	96.8	97.9	1.1
*BM EXECUTION*	100.0	100.0	0.0	92.9	97.1	4.2	96.3	98.7	2.4
*KI-67*	99.4	99.4	0.0	81.9	89.4	7.5	89.8	94.2	4.4

ARGO: Automatic Record Generator for Onco-hematology, OCR: Optical Character Recognition, NLP: Natural Language Processing, IHC: Immunohistochemistry, FISH: Fluorescent in situ Hybridization, BM: Bone Marrow, Diff: difference.

## Discussion

In the study, we aimed at designing a pipeline to automate the collection of RW onco-hematological data, about lymphoma diagnoses. Leveraging well-recognized technologies as OCR and NLP we developed a new tool, called ARGO, and provided a “proof” for its reliability in generating eCRFs directly from unstructured histopathology reports. We successfully tested ARGO performance, in terms of accuracy, precision, recall and F1 score, on a multicentric cohort of 326 lymphoma cases including DLBCL, FL, and MCL from seven independent centers.

ARGO generalizability stands on three assumptions: (1) the implementation of a function fully dedicated to recognize each input template independently of the clinical features collected in the report (a new template form an additional center might be easily read by adding few NLP regular expressions to the *header_function.py*); (2) ARGO is able to detect the set of clinically relevant terms for the diagnosis definition by matching standard criteria, that might be tailored to every clinical field (for example, other subtypes of lympho-proliferative disorders); (3) the choice at developing eCRFs on the College of American Pathologist (CAP) templates conferring high level of standardization to the clinical content.

In comparison with other applications in oncology, ARGO confirmed super-imposable performances in data field detection^6,11,14,16,21,22^, while overcoming some limitations. For instance, in the work by Nguyen et al., each metric decreases as the number of classes describing a certain data field increases^11^. This trend is globally confirmed in our experience, and even for data fields with high number of classes, such as “*SPECIMEN TYPE*”, we achieved a very high precision level. Also, to potentiate the OCR performance, we created three separate thesauri for “*BIOMARKERS*”, “*SPECIMEN TYPE*” and “*DIAGNOSIS*”. As shown in Tanenblatt et al.^22^, we first included officially-recognized nomenclatures in the “biomarkers” and “diagnosis” dictionaries, referring to the “International Statistical Classification of disease and related health problems 10th revision” (ICD10, version 2019, World Health Organization) classification^23^. Then, we manually added synonyms, abbreviations and other uncommon expressions noticed in our set of reports. Nevertheless, ARGO failed in converting six reports as a direct consequence of OCR-based limitations in reading reports with both low-quality optical resolution and describing IHC analyses from multiple samples. At this regard, the improvement of ARGO performance observed excluding OCR from the pipeline indicates a potential pitfall, which can be easily overcome by a manual supervision by a dedicated data-entry/manager.

From a more applicative point of view, ARGO might maximize the use of clinical data in translational research by boosting the adoption of EHR. Especially in onco-hematology, the public healthcare system still lacks standardized models of RW data collection, and several gaps exist concerning how to electronically collect unstructured information. Application of a computerized approach to extract data from paper-based reports and directly populate eCRFs provides two main advantages, such as the standardization of data collection and the data integration between Institutions and research networks. Finally, our system takes advantage from two levels of personalization related to REDCap, i) the designing of graphic interfaces directly by the clinical investigators according to specific clinical endpoints; and ii) the easily population of eCRFs via Application Programming Interface (API). Therefore, ARGO appeared as a valid tool for a precise and time-saving recording of clinical data when compared to manual abstraction^16^. Our approach results feasible in the daily practice, facilitating consultation, filtering, and management of RW data. This step is crucial to study wide proportions of onco-hematological patients who have no access to clinical trials and support national research networking.

Main limitations of the study could be the language of histopathology reports. However, current pathology reporting systems allow the use of personalized data fields according to shared templates and translating software, e.i. as “MyMemory” software, enable the easy switch up across languages. Moreover, providing the set of regular NLP rules used into ARGO might easily address this issue simply translating from Italian to other languages all words researched in the text included in each report.

Given the accuracy and efficiency in generating correct electronic records for multicentric subsets of different lymphoma types, our approach could be tailored to additional disease models in oncology and could set the basis to validate novel biomarkers for translational research.

## Methods

### Data collection

Overall, 332 histopathology paper-based reports were collected between 2014 and 2020 at the Pathology Unit of the IRCCS Istituto Tumori 'Giovanni Paolo II' in Bari, Italy (239) and from six different Italian centers (93) from Unit of Hematology, Azienda Ospedaliero-Universitaria Policlinico Umberto I in Rome, Italy, Hematology, AUSL/IRCCS of Reggio Emilia in Reggio Emilia, Italy, Division of Hematology 1, AOU “Città della Salute e della Scienza di Torino” in Turin, Italy, Division of Hematology, Azienda Ospedaliero-Universitaria Maggiore della Carità di Novara in Novara, Italy, Department of Medicine, Section of Hematology, University of Verona in Verona, Italy, and Division of Diagnostic Haematopathology, IRCCS European Institute of Oncology in Milan, Italy. The internal series included 106 DLBCL, 79 FL, and 54 MCL, while the external one comprised 49 DLBCL, 24 FL, and 20 MCL.

A unique ID code was assigned to each report. According to the diagnostic criteria for each lymphoma subtype, reports included IHC results obtained from LN, EN, BM or PB specimens. Qualitative and quantitative information for IHC markers including MYC, BCL2, BCL6, CD10, CD20, Cyclin-D1 were reported. Some reports also included molecular data from FISH analysis, while some reports included either FISH results or the level tumor cell infiltration as addendum. For DLBCL, molecular classification according to the COO estimated by the Hans algorithm was also included^24^. *Ki-67* proliferation index was also reported as quantitative value ranging from 5 to 100%.

The work was approved by the Institutional Review Board of the IRCCS Istituto Tumori “Giovanni Paolo II” hospital in Bari, Italy. All methods were carried out in accordance with relevant local regulations and after obtainment of dedicated informed consent.

### Automated detection of relevant terms in paper-based reports

We aimed this step of the workflow at automating the detection of relevant terms to be extracted from the text fields of paper-based reports. ARGO exploits OCR^25^ and NLP^26^ techniques to convert images of reports into text and detect relevant words in the text based on an “ad-hoc” thesaurus.

The conversion from image to text has been implemented in Tesseract OCR^©^ (version 4.1.1-rc2-20-g01fb). To improve conversion performance, each pathology report was firstly converted from pdf to image through Poppler library (version 0.26.5). Then, the image was translated in a grey scale of 8 bits (from 0 to 255 levels of grey).

Image transformation was developed in Python by OpenCV© software (version 4.2.0).

In ARGO, NLP techniques were adopted to automatically extract relevant terms for the disease diagnosis, to be transferred into the digitalized eCRFs. Thus, a set of NLP regular expressions were applied to extract information concerning the diagnosis, date of the report, report ID, type of the specimen, execution of BM biopsy, IHC, and FISH analyses, as well as quantitative and qualitative data of selected IHC markers (*MYC, BCL2, BCL6, CD10, CD20, Cyclin-D1*), *COO* subtypes and *Ki-67* proliferation index (paragraph “*ARGO function and NLP rules*”).

The disease nomenclature was assigned based on the highest match between the pattern of detected biomarkers in each report and a reference pattern, as reported in the “Hematopoietic and Lymphoid Neoplasm Coding Manual guidelines” from the “Surveillance, Epidemiology and End Results (SEER) program” of the National Institute of Health^27^. The final diagnosis nomenclature was referred to the ICD10 classification^23^. Communication between ARGO and SEER official servers was flexibly dealt via API.

ARGO was developed in Flask^©^, version 1.1.2, the webserver was an Oracle^©^ Linux Server 7.8 with kernel 4.14.35–1902.303.5.3.el7uek.x86_64. We used MariaDB^©^ 5.5.68 as database. NLP algorithms were developed in Python 3.6.8. Translation from English to Italian language was dealt via API tool MyMemory^©^ (version 3.5.0). To increase the detectability of biomarkers in the reports we also built three thesauri in Phyton with NLP regular expressions (Supplementary Appendix Source code S1 and Table S2). Despite the domain specificity of such thesauri, the technique of knowledge extraction by flexibly introducing a new thesaurus is a general feature of ARGO.

### ARGO functions and NLP rules

ARGO was developed according to three functions: *function_read.py*, *header_info.py*, and *params.py*. *Function_read.py* was the main function and incorporated (1) the call to the *header_info.py* function to recognize the report template as input, (2) the set of NLP expressions to identify both biomarker and diagnosis description, and (3) the call to the *params.py* function which included two API tokens, the first to take data on biomarkers and diagnosis from the SEER database and the second provided from the REDCap project ID to allow automatic data entry. Supplementary Fig. S2A details the pseudocode to process a pathology report. ARGO embedded two main activities, namely i) the recognition of the template from the header section including the fields “*BIOPSY DATE”* and “*ID NUMBER”,* the demographical patient information (“*NAME”*, “*SURNAME”, “DATE OF BIRTH”, “PLACE OF BIRTH”, “SEX”,* and “*SSN”* [Social Security Number]), and the “*SPECIMEN TYPE”* (via *header_info.py*), and ii) the recognition of the “*IHC MARKERS”* (“POSITIVITY/NEGATIVITY” or “QUANTITY”) from the biological samples, the fields “*FISH”*, “*DIAGNOSIS”,* and “*CELL OF ORIGIN”* from the disease section (via *function_read.py*). Supplementary Fig. S2B shows an example of NLP input from the internal series. The regular expressions used to automatically recognize the header section for internal reports are reported in Table 4. Those for the external reports are detailed in Supplementary Table S3.

**Table 4:** Set of NLP regular expressions embedded into the *header_function.py* for the internal reports.

	REDCap data label	*BIOPSY DATE*	*ID NUMBER*	*SURNAME*	*NAME*	*DATE OF BIRTH*	*PLACE OF BIRTH*	*SEX*	*SSN*	*SPECIMEN TYPE*
	REDCap data variable	nod_date_exam_req	nod_exam_num_req	pts_surname_demo	pts_name_demo	dob_demo	city_born_demo	sex_demo	ssn_demo	ln_specimen_dis
REPORT TEMPLATE for internal reports	Internal	“Accettazione” or ”Pervenuto” or “Richiesta” del” or “Ricevimento”	"N. Esame"	"Cognome"	"Nome"	"Data di nascita"	"Comune di Nascita"	"Sesso"	"Codice Fiscale"	"Materiale Inviato"
	NLP pattern	cettaz. +|ervenuto. +|ichiesta.*del. + [0–3][0–9]/[0–1][0–9]/2[0–9][0–9][0–9]	+ same. *[0–3][0–9-.-d]	COGNOME.*|COGNOME.*DATA|COGNOME.*CITT	\\bNOME.*|\\bNOME.*DATA|\\bNOME.*CITT	. + asci. + [0–3][0–9]/[0–1][0–9]/[1, 2][0–9][0–9][0–9]	. + omu. + asci. + \w +	. + ess.{1,3}m	[A-Z]{6}[0–9][0–9][A-Z][0–9]{2}[A-Z][0–9]{3}[A-Z]	ate. + al. + via. + \n. +

NLP: Natural Language Processing; ID: Identification; NA, Not Available, SSN, Social Security Number.

Concerning *function_read.py*, we identified the set of pathological description patterns according to the following four scenarios:

1. description of qualitative markers by symbolic qualifiers in a free text form (e.g. “ + ” for positivity and “-” for negativity);
2. description of qualitative markers by textual qualifiers in a free text form (e.g. “positive”, “reactive” or “immunoreactive” for positivity and “negative” or “immunonegative” for negativity);
3. description of both qualitative and quantitative markers by symbolic or textual qualifiers in a bullet form;
4. description of pure quantitative markers (as *Ki-67*).

Table 5 shows three representative patterns of description with their relative NLP pseudocodes and expected results. The whole set of patterns is detailed in Supplementary Table S4.

**Table 5:**
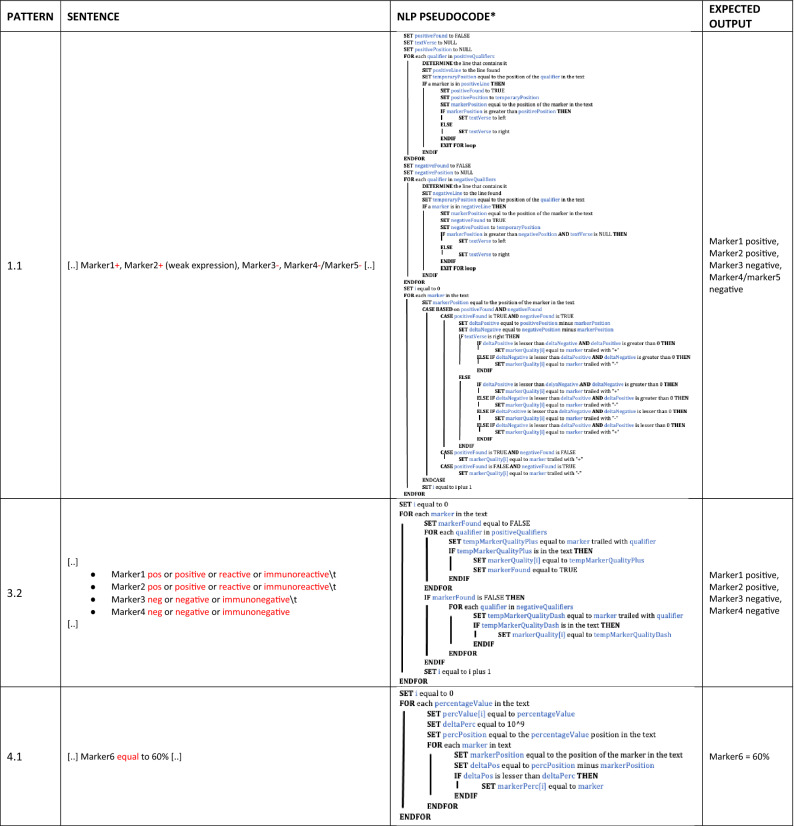
Representative sets of NLP rules embedded into the function_read.py for patterns 1.1, 3.2, and 4.1.

*Precondition. For each case we,

REMOVE SPACES;

REMOVE NEWLINES;

TRANSFORM "." in NEWLINES;

positiveQualifiers = [pos, positive, reactive, immunoreactive];

negativeQualifiers = [neg, negative, immunonegative].

Abbreviations. NLP: Natural Language Processing.

### Data-mapping and automatic population of eCRFs

For a systematic collection of the diagnostic variables in this study, we designed dedicated eCRFs on REDcap^17,18^. eCRFs were suited to the synoptic templates provided and approved by the CAP. We referred to DLBCL, FL, and MCL templates^28,29^. The data-mapping between ARGO and the eCRFs was performed by providing the relevant data fields from the REDCap dictionary as a flexible input to the application (Supplementary Table S5). Finally, we used API technology for the automatic data entry and final upload of the information of interest into the eCRFs.

### Validation metrics

ARGO performance, regarded as the level of consistency between data included in the original pathology reports and those automatically transferred into eCRFs, was assessed in terms of accuracy, precision, recall and F1 score^30^. To calculate each measure, we defined the cases in the following (1) *true-positive*: cases in which ARGO detected correctly the expected variables; (2) *false-positive*: cases in which ARGO detected variables even if not present in the original report; (3) *true-negative*: cases in which ARGO did not detect a variable not present in the original report; and (4) *false-negative*: cases in which ARGO failed in detecting a variable present in the original report.

Results for each data-field of internal and external series were statistically compared by a chi-square test.

## Supplementary Information

Supplementary Information 1.
