# Supplementary material for: Electronic case report forms generation from pathology reports by ARGO, automatic record generator for onco-hematology
Source: Sci Rep. 2021 Dec 10;11:23823. doi: 10.1038/s41598-021-03204-z (PMC8664934; doi:10.1038/s41598-021-03204-z)
Supplement: Supplementary file 1 — Supplementary Information 1. [file 41598_2021_3204_MOESM1_ESM.pdf]

Electronic Case Report Forms generation from pathology reports by ARGO, Automatic Record Generator for Onco-hematology.

SUPPLEMENTARY APPENDIX

- **Figure S1**. Figure 1B from main manuscript in high resolution format.

- **Figure S2**. Logical description of the NLP rules implemented in ARGO.

- **Table S1**. Performance metric from n. 239 internal and n. 93 external pathology reports.

- **Table S2**. Referred thesaurus for biomarkers recognition and for the diagnosis definition.

- **Table S3**. Set of NLP regular expressions embedded into the *header_function.py*.

- **Table S4**. Set of NLP rules embedded into the *function_read.py* for the whole patterns identified according to each scenario.

- **Table S5**. Data dictionary extracted from REDCap for data fields used to map each word detected from the NLP.

- **Source code S1.** Source code developed in Python for the application of the thesaurus rules.

- **References**

This supplementary material has been provided by the authors to give readers additional information about their work.

Figure S1. Figure 1B from main manuscript in high resolution format.

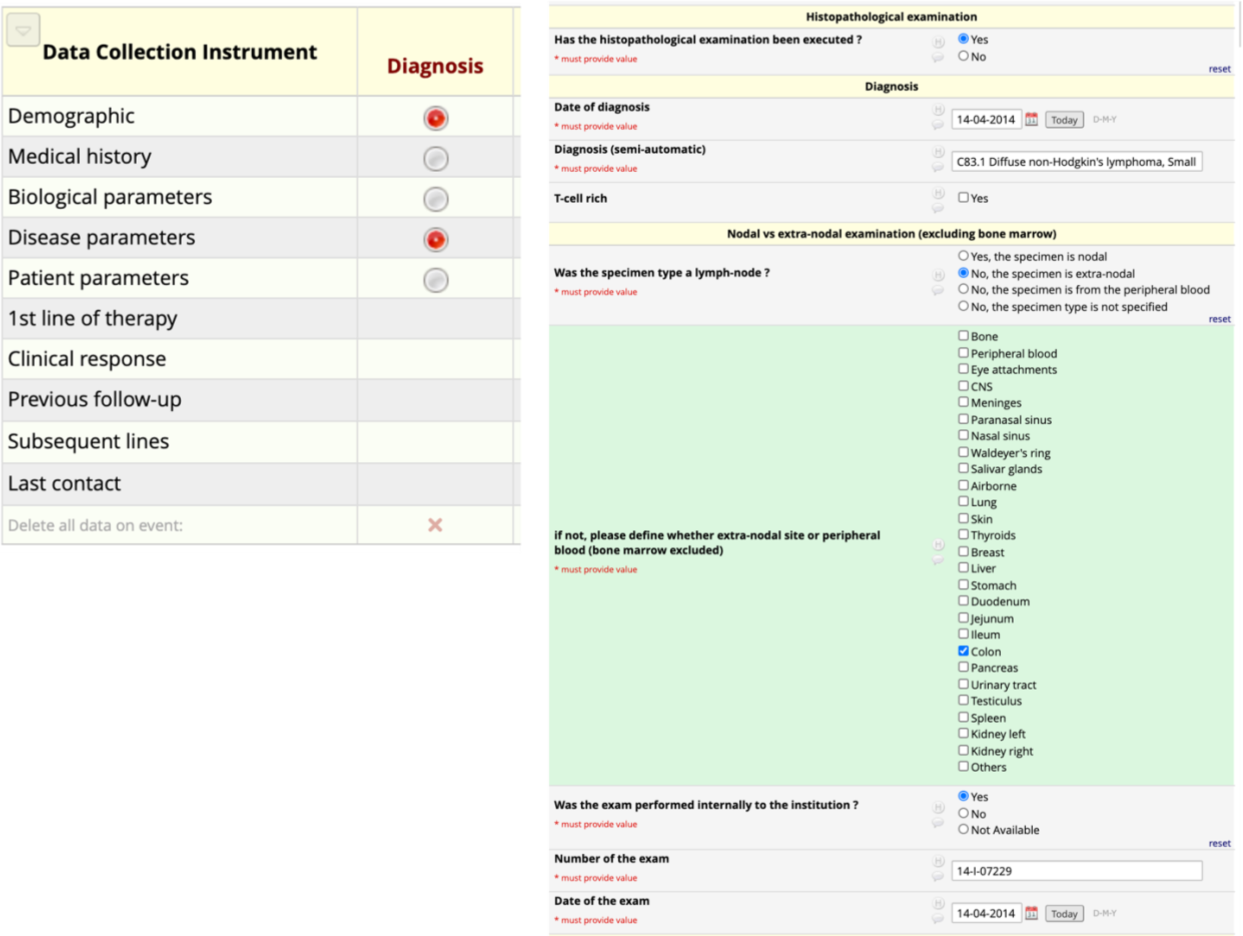

Representative picture of REDCap dashboard for a single case report including “Demography” and “Disease parameters” forms (red bullets).

Figure S2. Logical description of the NLP rules implemented in ARGO.

A

header_info.py

1) ARGO recognized the hospital template in the header section (NLP regular expression reported in Supplementary Table S1). Thus,

1.1) ARGO sought words related to the reported date to initialize the *BIOPSY DATE* data-field.

1.2) ARGO sought words related to the report ID date to initialize the *the ID NUMBER* data-field

1.3) ARGO sought the words related to the patient's identification (e.g. "Cognome", "Nome"):

- *NAME, SURNAME, DATE OF BIRTH, PLACE OF BIRTH data fields*

- *SSN*,
  i. in case the SSN code was present, ARGO initialized the SSN code data-field,
  ii. In case the SSN code was not present, ARGO automatically calculated the SSN code via external webservice from *NAME*, *SURNAME, DATE OF BIRTH, PLACE OF BIRTH* data-fields.

1.4) ARGO sought the words related to the specimen material at identifying the *SPECIMEN TYPE* data-field

function_read.py

A. IHC MARKERS. For each marker recognized in the text (Supplementary Table S2)
  i. ARGO prompted the biomarker to the SEER database via API key (via *params.py*),
  ii. The SEER database responded providing the relative biomarker,
  iii. if either points i) or ii) failed, ARGO internally prompted the biomarker to the "in-house" thesaurus (Supplementary Table S1);
  iv. If also the point iii) failed, the relative data field from eCRF was not initialized.

B. IHC MARKERS (POSITIVITY/NEGATIVITY). A marker was assumed positive if the nearest adjective/noun reported on the left was "positivo/positività" or, if appended to marker is reported a '+' (plus). A marker was assumed negative if the nearest adjective/noun reported on the left is "negativo/negatività" or, if appended to marker was reported a '-' (dash).

C. IHC MARKERS (QUANTITY). Markers expressed with a quantity in percentage (e.g. Ki-67, MYC, BCL2, BCL6) were identified if the nearest marker on the left was a percentage number, as expected.

D. FISH.
  i. ARGO sought if the FISH exam was added,
  ii. ARGO sought FISH markers (MYC, BCL2, BCL6, and CYCLIN D1) and if they were either positive or negative. A marker was assumed positive if the nearest adjective/noun reported on the left was "positivo/positività" or, if appended to marker was reported a '+' (plus). A marker was assumed negative if the nearest adjective/noun reported on the left was "negativo/negatività" or, if appended to marker was reported a '-' (dash).

E. CELL OF ORIGIN. ARGO seeks in the report the words "Germinal Center B-like" or "GCB". The COO is assumed negative if the nearest word reported on the left of the COO is "non" or "no".

F. DIAGNOSIS.
  i. ARGO prompted the diagnosis to the SEER database via API key (via *params.py*),
  ii. The SEER database responded providing the relative biomarker,
  iii. if either points i) or ii) failed, ARGO internally prompted the diagnosis to the "in-house" thesaurus (Supplementary Table S1);
  iv. If also the point iii) failed, the relative diagnosis from eCRF was not initialized.

B

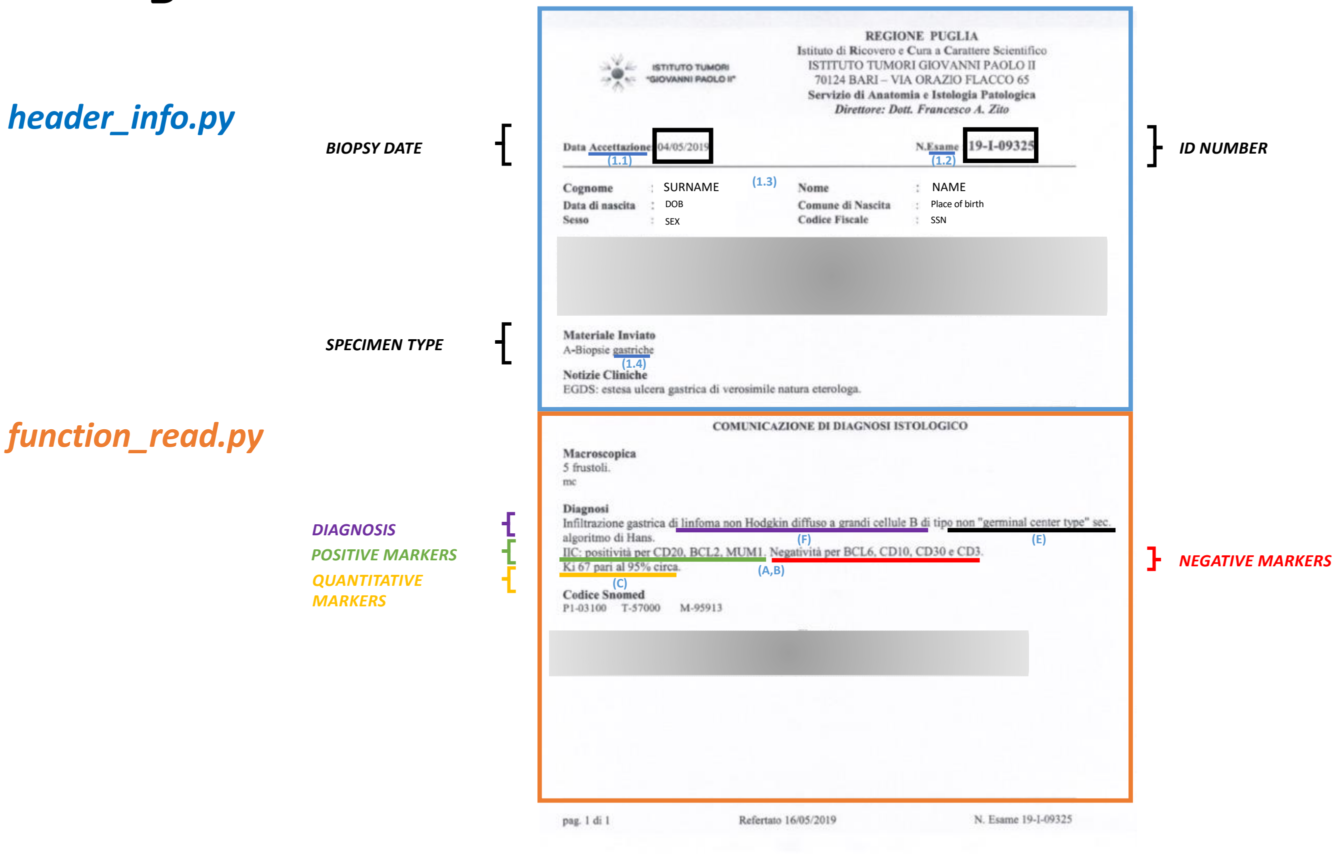

A) The pseudocode describes all logical phases executed by ARGO in recognizing each data field from the header and disease section of a paper-based report. **B)** Application of each NLP phase on an example of paper-based report from the internal series (Pathology Unit of the IRCCS Istituto Tumori "Giovanni Paolo II" of Bari, Italy).

Abbreviations. ARGO: Automatic Record Generator for Onco-hematology, NLP: Natural Language Processing, ID: Identification, SSN: security social number, SEER: Surveillance, Epidemiology, and End Result API: Application Programming Interface, IHC: Immunohistochemistry, FISH: Fluorescent in situ hybridization, COO: cell of origin, GCB: Germinal Center B-like.

Table S1. Performance metric from n. 239 internal and n. 93 external pathology reports.

DATA FIELD	INTERNAL SERIES				EXTERNAL SERIES				p
	Accuracy	Precision	Recall	F1-score	Accuracy	Precision	Recall	F1-score	
DIAGNOSIS	87.9	100.0	87.9	93.5	88.2	100.0	88.2	93.7	1.000
BIOPSY DATE	97.1	100.0	97.1	98.5	94.6	100.0	90.6	95.0	0.8833
ID NUMBER	92.1	100.0	92.1	95.9	83.9	100.0	77.3	87.2	0.2134
SPECIMEN TYPE	86.6	98.5	87.2	92.5	91.4	100.0	91.4	95.5	0.9063
IHC EXECUTION	95.4	100.0	95.4	97.6	97.8	100.0	97.8	98.9	0.9868
FISH EXECUTION	93.7	100.0	93.7	96.8	98.9	100.0	98.9	99.5	0.8918
BM EXECUTION	92.9	100.0	92.9	96.3	96.8	100.0	96.8	98.4	0.9494
COO	96.2	97.2	81.4	88.6	94.6	100.0	84.8	91.8	0.9491
MYC	93.7	100.0	57.1	72.7	89.2	96.7	76.3	85.3	0.0716
BCL2	77.4	98.5	71.4	82.8	82.8	98.4	80.8	88.7	0.6090
BCL6	69.5	99.1	61.5	75.9	79.6	98.1	74.3	84.6	0.2231

CD10	67.4	96.9	55.9	70.9	82.8	98.5	81.0	88.9	0.0025
CD20	78.2	99.4	76.3	86.3	91.4	100.0	90.7	95.1	0.1715
CYCLIN D1	90.4	100.0	71.6	83.5	86.0	100.0	69.8	82.2	0.9622
KI-67	85.4	99.4	81.9	89.8	80.6	100.0	76.3	86.6	0.8469

Abbreviations. IHC, immunohistochemistry; FISH, fluorescent *in situ* hybridization; BM, bone marrow; CD, cluster of differentiation; COO, cell of origin subtype.

Table S2. Referenced thesaurus for biomarkers recognition and for the diagnosis definition.

DIAGNOSIS[1, 2]	MARKER	TYPE	VARIANTS					
C83.3 DIFFUSE NON-HODGKINS LYMPHOMA, LARGE CELL (DIFFUSE)	BCL2 expression	Immunophenotyping	BCL-2	bcl2	bcl 2			
C83.3 DIFFUSE NON-HODGKINS LYMPHOMA, LARGE CELL (DIFFUSE)	MYC expression	Immunophenotyping	myc	C-MYC	c-myc			
C83.3 DIFFUSE NON-HODGKINS LYMPHOMA, LARGE CELL (DIFFUSE)	BCL6 positive	Immunophenotyping	bcl6	BCL-6				
C83.3 DIFFUSE NON-HODGKINS LYMPHOMA, LARGE CELL (DIFFUSE)	CD5 expression	Immunophenotyping	cd5	cd 5	CD 5			
C83.3 DIFFUSE NON-HODGKINS LYMPHOMA, LARGE CELL (DIFFUSE)	CD10 positive	Immunophenotyping	cd10	cd 10	CD 10			
C83.3 DIFFUSE NON-HODGKINS LYMPHOMA, LARGE CELL (DIFFUSE)	CD15 positive	Immunophenotyping	cd15	cd 15	CD 15			
C83.3 DIFFUSE NON-HODGKINS LYMPHOMA, LARGE CELL (DIFFUSE)	CD19 expression	Immunophenotyping	cd19	cd 19	CD 19			
C83.3 DIFFUSE NON-HODGKINS LYMPHOMA, LARGE CELL (DIFFUSE)	CD20 expression	Immunophenotyping	cd20	cd 20	CD 20			
C83.3 DIFFUSE NON-HODGKINS LYMPHOMA, LARGE CELL (DIFFUSE)	CD22 expression	Immunophenotyping	cd22	cd 22	CD 22			
C83.3 DIFFUSE NON-HODGKINS LYMPHOMA, LARGE CELL (DIFFUSE)	CD30 expression	Immunophenotyping	cd30	cd 30	CD 30			
C83.3 DIFFUSE NON-HODGKINS LYMPHOMA, LARGE CELL (DIFFUSE)	CD79a expression	Immunophenotyping	cd79a	cd 79a	CD 79a			
C83.3 DIFFUSE NON-HODGKINS LYMPHOMA, LARGE CELL (DIFFUSE)	FOXP1 expression	Immunophenotyping	foxp1					
C83.3 DIFFUSE NON-HODGKINS LYMPHOMA, LARGE CELL (DIFFUSE)	IRF4/MUM1 positive	Immunophenotyping	MUM1/IRF4	MUM-1/IRF4	MUM 1/IRF4	MUM1-IRF4	MUM-1-IRF4	MUM 1-IRF4
C83.3 DIFFUSE NON-HODGKINS LYMPHOMA, LARGE CELL (DIFFUSE)	LMO2 expression	Immunophenotyping	lmo2	LMO 2	lmo 2			
C83.3 DIFFUSE NON-HODGKINS LYMPHOMA, LARGE CELL (DIFFUSE)	PAX5 expression	Immunophenotyping	pax5	PAX 5	pax 5			
C82.9 FOLLICULAR NON-HODGKIN'S LYMPHOMA, UNSPECIFIED	BCL2 expression and positive	Immunophenotyping	BCL-2	bcl2	bcl 2			

C82.9 FOLLICULAR NON-HODGKIN'S LYMPHOMA, UNSPECIFIED	BCL6 positive	Immunophenotyping	bcl6	BCL-6		
C82.9 FOLLICULAR NON-HODGKIN'S LYMPHOMA, UNSPECIFIED	CD5 negative	Immunophenotyping	cd5	cd 5	CD 5	
C82.9 FOLLICULAR NON-HODGKIN'S LYMPHOMA, UNSPECIFIED	CD10 expression and positive	Immunophenotyping	cd10	cd 10	CD 10	
C82.9 FOLLICULAR NON-HODGKIN'S LYMPHOMA, UNSPECIFIED	CD19 expression	Immunophenotyping	cd19	cd 19	CD 19	
C82.9 FOLLICULAR NON-HODGKIN'S LYMPHOMA, UNSPECIFIED	CD20 positive	Immunophenotyping	cd20	cd 20	CD 20	
C82.9 FOLLICULAR NON-HODGKIN'S LYMPHOMA, UNSPECIFIED	CD22 expression	Immunophenotyping	cd22	cd 22	CD 22	
C82.9 FOLLICULAR NON-HODGKIN'S LYMPHOMA, UNSPECIFIED	CD23 expression	Immunophenotyping	cd23	cd 23	CD 23	
C82.9 FOLLICULAR NON-HODGKIN'S LYMPHOMA, UNSPECIFIED	CD43 negative	Immunophenotyping	cd43	cd 43	CD 43	
C82.9 FOLLICULAR NON-HODGKIN'S LYMPHOMA, UNSPECIFIED	CD79a expression and positive	Immunophenotyping	cd79a	cd 79a	CD 79a	
C82.9 FOLLICULAR NON-HODGKIN'S LYMPHOMA, UNSPECIFIED	GCET1 positive	Immunophenotyping	gcet1	CGET 1	cget 1	
C82.9 FOLLICULAR NON-HODGKIN'S LYMPHOMA, UNSPECIFIED	GCET2 (HGAL) positive	Immunophenotyping	cget2	CGET 2	cget 2	
C82.9 FOLLICULAR NON-HODGKIN'S LYMPHOMA, UNSPECIFIED	IgD negative	Immunophenotyping	igd	Igd	igD	IGD
C82.9 FOLLICULAR NON-HODGKIN'S LYMPHOMA, UNSPECIFIED	IgM positive	Immunophenotyping	igm	Igm	igM	IGM
C82.9 FOLLICULAR NON-HODGKIN'S LYMPHOMA, UNSPECIFIED	LMO2 positive	Immunophenotyping	lmo2	LMO 2	lmo 2	
C82.9 FOLLICULAR NON-HODGKIN'S LYMPHOMA, UNSPECIFIED	PAX5 positive	Immunophenotyping	pax5	PAX 5	pax 5	
C83.1 DIFFUSE NON-HODGKIN'S LYMPHOMA, SMALL CLEAVED CELL (DIFFUSE)	BCL2 positive	Immunophenotyping	BCL-2	bcl2	bcl 2	
C83.1 DIFFUSE NON-HODGKIN'S LYMPHOMA, SMALL CLEAVED CELL (DIFFUSE)	BCL6 negative	Immunophenotyping	bcl6	BCL-6		

C83.1 DIFFUSE NON-HODGKIN'S LYMPHOMA, SMALL CLEAVED CELL (DIFFUSE)	CD5 positive	Immunophenotyping	cd5	cd 5	CD 5		
C83.1 DIFFUSE NON-HODGKIN'S LYMPHOMA, SMALL CLEAVED CELL (DIFFUSE)	CD10 negative	Immunophenotyping	cd10	cd 10	CD 10		
C83.1 DIFFUSE NON-HODGKIN'S LYMPHOMA, SMALL CLEAVED CELL (DIFFUSE)	CD23 negative or weakly positive	Immunophenotyping	cd23	cd 23	CD 23		
C83.1 DIFFUSE NON-HODGKIN'S LYMPHOMA, SMALL CLEAVED CELL (DIFFUSE)	CD43 positive	Immunophenotyping	cd43	cd 43	CD 43		
C83.1 DIFFUSE NON-HODGKIN'S LYMPHOMA, SMALL CLEAVED CELL (DIFFUSE)	Cyclin D1 expression	Immunophenotyping	cyclin D1	cyclin D1	CYCLIN D1	Cyclin D1	
C83.1 DIFFUSE NON-HODGKIN'S LYMPHOMA, SMALL CLEAVED CELL (DIFFUSE)	FMC7 positive	Immunophenotyping	fmc7	FMC 7	fmc 7		
C83.1 DIFFUSE NON-HODGKIN'S LYMPHOMA, SMALL CLEAVED CELL (DIFFUSE)	IgM/IgD expression	Immunophenotyping	igm/igd	IGM/IGD	Igm/Igd		
C83.1 DIFFUSE NON-HODGKIN'S LYMPHOMA, SMALL CLEAVED CELL (DIFFUSE)	IRF4 positive	Immunophenotyping	irf4	IRF 4	irf 4		
C83.1 DIFFUSE NON-HODGKIN'S LYMPHOMA, SMALL CLEAVED CELL (DIFFUSE)	MUM1 positive	Immunophenotyping	MUM-1	MUM 1	mum1	mum-1	mum 1

Abbreviations. CD, cluster of differentiation; Ig, immunoglobulins.

Table S3. Set of NLP regular expressions embedded into the *header_function.py*.

<table>
<tr><td></td><td>REDCAP data label</td><td>BIOPSY DATE</td><td>ID NUMBER</td><td>SURNAME</td><td>NAME</td><td>DATE OF BIRTH</td><td>PLACE OF BIRTH</td><td>SEX</td><td>SSN</td><td>SPECIMEN TYPE</td></tr>
<tr><td></td><td>REDCAP data variable</td><td>nod_date_exam_req</td><td>nod_exam_num_req</td><td>pts_surname_demo</td><td>pts_name_demo</td><td>dob_demo</td><td>city_born_demo</td><td>sex_demo</td><td>ssn_demo</td><td>ln_specimen_dis</td></tr>
<tr><td rowspan="2">REPORT TEMPLATE 1</td><td>Internal</td><td>“Accettazione” or ”Pervenuto” or “Richiesta” del” or “Ricevimento”</td><td>"N. Esame"</td><td>"Cognome"</td><td>"Nome"</td><td>"Data di nascita"</td><td>"Comune di Nascita"</td><td>"Sesso"</td><td>"Codice Fiscale"</td><td>"Materiale Inviato"</td></tr>
<tr><td>NLP pattern</td><td>cettaz.+|ervenuto.+|ichiesta.*del.+ [0-3][0-9]/[0-1][0-9]/2[0-9][0-9][0-9]</td><td>.+same.*[0-3][0-9]-.-\d+</td><td>COGNOME.*|COGNOME.*DATA|COGNOME.*CITT</td><td>\\bNOME.*|\\bNOME.*DATA|\\bNOME.*CITT</td><td>.+asci.+[0-3][0-9]/[0-1][0-9]/[1-2][0-9][0-9][0-9]</td><td>.+omu.+asci.+\w+</td><td>.+ess.{1,3}m</td><td>[A-Z]{6}[0-9][0-9][A-Z][0-9]{2}[A-Z][0-9]{3}[A-Z]</td><td>ate.+al.+via.+\n.+</td></tr>
<tr><td rowspan="2">REPORT TEMPLATE 2</td><td>External</td><td>“Accettazione” or ”Pervenuto” or “Richiesta” del” or “Ricevimento”</td><td>"Esame ISTOLOGICO N."</td><td>NA</td><td>NA</td><td>NA</td><td>NA</td><td>NA</td><td>NA</td><td>"Materiale Inviato"</td></tr>
<tr><td>NLP pattern</td><td>cettaz.+|ervenuto.+|ichiesta.*del.+ [0-3][0-9]/[0-1][0-9]/2[0-9][0-9][0-9]</td><td>.+same.*[0-9][0-9][0-9][0-9]/.+/.</td><td>NA</td><td>NA</td><td>NA</td><td>NA</td><td>NA</td><td>NA</td><td>ate.+al.+via.+\n.+|ate.+al.+via.+\n\n.+|izie.+inich.+\n.+|izie.+inich.+\n\n.+|ate.+al.+via.+izie.+inich</td></tr>
<tr><td rowspan="2">REPORT TEMPLATE 3</td><td>External</td><td>“Accettazione” or ”Pervenuto” or “Richiesta” del” or “Ricevimento”</td><td>"ESAME ISTOLOGICO"</td><td>NA</td><td>NA</td><td>NA</td><td>NA</td><td>NA</td><td>NA</td><td>"NOTIZIE CLINICHE"</td></tr>
<tr><td>NLP pattern</td><td>cettaz.+|ervenuto.+|ichiesta.*del.+ [0-3][0-9]/[0-1][0-9]/2[0-9][0-9][0-9]</td><td>SAM.*[0-9][0-9]/.+</td><td>NA</td><td>NA</td><td>NA</td><td>NA</td><td>NA</td><td>NA</td><td>izie.+inich.+stologica</td></tr>
</table>

<table>
<tr><td rowspan="2">REPORT TEMPLATE 4</td><td>External</td><td>“Accettazione” or ”Pervenuto” or “Richiesta” del” or “Ricevimento”</td><td>"Esame"</td><td>NA</td><td>NA</td><td>NA</td><td>NA</td><td>NA</td><td>NA</td><td>"Materiale Inviato"</td></tr>
<tr><td>NLP pattern</td><td>cettaz.+|ervenuto.+|ichiesta.*del.+ [0-3][0-9]/[0-1][0-9]/2[0-9][0-9][0-9]</td><td>SAM.*[0-9][0-9].+</td><td>NA</td><td>NA</td><td>NA</td><td>NA</td><td>NA</td><td>NA</td><td>ate.+al.+via.+\n.+</td></tr>
<tr><td rowspan="2">REPORT TEMPLATE 5</td><td>External</td><td>“Accettazione” or ”Pervenuto” or “Richiesta” del” or “Ricevimento”</td><td>"N. esame"</td><td>NA</td><td>NA</td><td>NA</td><td>NA</td><td>NA</td><td>NA</td><td>"MATERIALE INVIATO"</td></tr>
<tr><td>NLP pattern</td><td>cettaz.+|ervenuto.+|ichiesta.*del.+ [0-3][0-9]/[0-1][0-9]/2[0-9][0-9][0-9]</td><td>.+same.*[0-3][0-9]-.-\d+</td><td>NA</td><td>NA</td><td>NA</td><td>NA</td><td>NA</td><td>NA</td><td>ate.+al.+via.+\n.+</td></tr>
<tr><td rowspan="2">REPORT TEMPLATE 6</td><td>External</td><td>"Data"</td><td>"Esame"</td><td>NA</td><td>NA</td><td>NA</td><td>NA</td><td>NA</td><td>NA</td><td>"Sede/Materiale in esame"</td></tr>
<tr><td>NLP pattern</td><td>ata.+</td><td>SAM.*[0-9][0-9].+</td><td>NA</td><td>NA</td><td>NA</td><td>NA</td><td>NA</td><td>NA</td><td>ate.+al.+esa.+\n.+\n.+</td></tr>
<tr><td rowspan="2">REPORT TEMPLATE 7</td><td>External</td><td>“Accettazione” or ”Pervenuto” or “Richiesta” del” or “Ricevimento”</td><td>"Esame"</td><td>NA</td><td>NA</td><td>NA</td><td>NA</td><td>NA</td><td>NA</td><td>“MATERIALE” or first line after “DESCRIZIONE MISCROSCOPICA</td></tr>
<tr><td>NLP pattern</td><td>cettaz.+|ervenuto.+|ichiesta.*del.+|ricevimento.+</td><td>SAM.*[0-9][0-9].+</td><td>NA</td><td>NA</td><td>NA</td><td>NA</td><td>NA</td><td>NA</td><td>aterial.+escrizione</td></tr>
</table>

Abbreviations. NLP, Natural Language Processing; ID, Identification; NA, Not Available, SSN, Social Security Number.

Table S4. Set of NLP rules embedded into the *function_read.py* for the whole patterns identified according to each scenario.

PATTERN	SENTENCE	NLP PSEUDOCODE*	EXPECTED OUTPUT
1.1	[..] Marker1+, Marker2+ (weak expression), Marker3-, Marker4-/Marker5- [..]	CASE A	Marker1 positive, Marker2 positive, Marker3 negative, Marker4/marker5 negative
1.2	[..] Marker1++, Marker2+/-, Marker3--, Marker4-/+ [..]	CASE A	Marker1 positive, Marker2 positive, Marker3 negative, Marker4/marker5 negative
1.3	[..] Marker1+, Marker2+ (weak expression), Marker3\t -, Marker4-/Marker5- [..]	CASE A	Marker1 positive, Marker2 positive, Marker3 negative, Marker4/marker5 negative
2.1	[..] pos or positive or reactive or immunoreactive markers are Marker1, Marker2. Neg or Negative or Immunonegative markers are Marker3, Marker 4 [..]	CASE B	Marker1 positive, Marker2 positive, Marker3 negative, Marker4 negative
2.2	[..] Marker1 pos or positive or reactive or immunoreactive, Marker2 pos or positive or reactive (weak expression), Marker3 neg or negative or immunonegative, Marker4/Marker5 neg or negative or immunonegative[..]	CASE B	Marker1 positive, Marker2 positive, Marker3 negative, Marker4 negative
2.3	[..] positivity or immunoreactivity or reactivity for Marker1, Marker2, negativity or immunonegativity for Marker3, Marker4 [..]	CASE B	Marker1 positive, Marker2 positive, Marker3 negative, Marker4 negative
2.4	[..] Marker1, Marker2, Marker3 pos or positive or immunoreactive or reactive, Marker4, Marker5 neg or negative or immunonegative[..]	CASE B	Marker 1 positive, Marker 2 positive, Marker 3 positive, Marker 4 negative, Marker 5 negative
2.5	[..] Marker1, Marker2, Marker3 positivity or immunoreactivity or reactivity, Marker4, Marker5 negativity or immunonegativity [..]	CASE B	Marker 1 positive, Marker 2 positive, Marker 3 positive, Marker 4 negative, Marker 5 negative
2.6	[..] positivity or immunoreactivity or reactivity for Marker1, Marker2,\t negativity or immunonegativity for Marker3, Marker4 [..]	CASE B	Marker1 positive, Marker2 positive, Marker3 negative, Marker4 negative
2.7	[..] positivity or immunoreactivity or reactivity for Marker1,\t Marker2, negativity or immunonegativity for Marker3, Marker4 [..]	CASE B	Marker1 positive, Marker2 positive, Marker3 negative, Marker4 negative
2.8	[..] pos or positive or reactive or immunoreactive markers are Marker1, Marker2.\t Neg or Negative or Immunonegative markers are Marker3, Marker 4 [..]	CASE B	Marker1 positive, Marker2 positive, Marker3 negative, Marker4 negative
2.9	[..] pos or positive or reactive or immunoreactive markers are Marker1,\t Marker2. Neg or Negative or Immunonegative markers are Marker3, Marker 4 [..]	CASE B	Marker1 positive, Marker2 positive, Marker3 negative, Marker4 negative
2.10	[..] Marker1, Marker2, Marker3 positivity or immunoreactivity or reactivity,\t Marker4, Marker5 negativity or immunonegativity [..]	CASE B	Marker 1 positive, Marker 2 positive, Marker 3 positive, Marker 4 negative, Marker 5 negative
2.11	[..] Marker1, Marker2,\t Marker3 positivity or immunoreactivity or reactivity, Marker4, Marker5 negativity or immunonegativity [..]	CASE B	Marker 1 positive, Marker 2 positive, Marker 3 positive, Marker 4 negative, Marker 5 negative
2.12	[..] Marker1 and Marker2 are pos or positive or immunoreactive or reactive and neg or negative or immunonegative respectively [..]	CASE B	Marker 1 positive, Marker 2 positive
2.13	[..] Marker1 and Marker2 \t are pos or positive or immunoreactive or reactive and neg or negative or immunonegative respectively [..]	CASE B	Marker 1 positive, Marker 2 positive
2.14	[..] Marker1 and Marker2 are pos or positive or immunoreactive or reactive and \t neg or negative or immunonegative respectively [..]	CASE B	Marker 1 positive, Marker 2 positive
3.1	[..]<br>• Marker1+\t<br>• Marker2+\t<br>• Marker3-\t	CASE A	Marker1 positive, Marker2 positive, Marker3 negative, Marker4 negative

	• Marker4-<br>[..]		
3.2	[..]<br>• Marker1 pos or positive or reactive or immunoreactive\t<br>• Marker2 pos or positive or reactive or immunoreactive\t<br>• Marker3 neg or negative or immunonegative\t<br>• Marker4 neg or negative or immunonegative<br>[..]	CASE C	Marker1 positive, Marker2 positive, Marker3 negative, Marker4 negative
3.3	[..]<br>Positive or Reactive or Immunoreactive markers:\t<br>• Marker1, Marker2\t<br>\t<br>Negative or Immunonegative markers:\t<br>• Marker3, Marker4<br>[..]	CASE B	Marker1 positive, Marker2 positive, Marker3 negative, Marker4 negative
3.4	[..]<br>Positive or Reactive or Immunoreactive markers:\t<br>• Marker1\t<br>• Marker2\t<br>\t<br>Negative or Immunonegative markers:\t<br>• Marker3\t<br>• Marker4<br>[..]	CASE B	Marker1 positive, Marker2 positive, Marker3 negative, Marker4 negative
3.5	[..]<br>Positivity or Reactivity or Immunoreactivity:\t<br>• Marker1, Marker2\t<br>\t<br>Negativity or Immunonegativity:\t<br>• Marker3, Marker4<br>[..]	CASE B	Marker1 positive, Marker2 positive, Marker3 negative, Marker4 negative
3.6	[..]<br>Positivity or Reactivity or Immunoreactivity:\t<br>Marker1\t<br>Marker2\t<br>\t<br>Negativity or Immunonegativity:\t<br>Marker3\t<br>Marker4<br>[..]	CASE B	Marker1 positive, Marker2 positive, Marker3 negative, Marker4 negative
4.1	[..] Marker6 equal to 60% [..]	CASE D	Marker6 = 60%
4.2	[..] Marker6 similar to 60% [..]	CASE D	Marker6 = 60%

4.3	[..] Marker6 = or > or < 60% [..]	CASE D	Marker6 = 60%
4.4	[..] Marker6 (60%) [..]	CASE D	Marker6 = 60%
4.5	[..] Marker6 between 30% and 60% [..]	CASE D	Marker6 = 60%
4.6	[..] Marker6 with sixty % of expression [..]	CASE D	Marker6 = NA
4.7	[..] low expression of Marker6 [..]	CASE D	Marker6 = NA

Abbreviations. NLP, Natural Language Processing.

Where, CASE A is:

```
SET positiveFound to FALSE
SET textVerse to NULL
SET positivePosition to NULL
FOR each qualifier in positiveQualifiers
|    DETERMINE the line that contains it
|    SET positiveLine to the line found
|    SET temporaryPosition equal to the position of the qualifier in the text
|    IF a marker is in positiveLine THEN
|    |    SET positiveFound to TRUE
|    |    SET positivePosition to temporaryPosition
|    |    SET markerPosition equal to the position of the marker in the text
|    |    IF markerPosition is greater than positivePosition THEN
|    |    |    SET textVerse to left
|    |    ELSE
|    |    |    SET textVerse to right
|    |    ENDIF
|    |    EXIT FOR loop
|    ENDIF
ENDFOR
SET negativeFound to FALSE
SET negativePosition to NULL
FOR each qualifier in negativeQualifiers
|    DETERMINE the line that contains it
|    SET negativeLine to the line found
|    SET temporaryPosition equal to the position of the qualifier in the text
|    IF a marker is in negativeLine THEN
|    |    SET markerPosition equal to the position of the marker in the text
|    |    SET negativeFound to TRUE
|    |    SET negativePosition to temporaryPosition
|    |    IF markerPosition is greater than negativePosition AND textVerse is NULL THEN
|    |    |    SET textVerse to left
|    |    ELSE
|    |    |    SET textVerse to right
|    |    ENDIF
|    |    EXIT FOR loop
|    ENDIF
ENDFOR
SET i equal to 0
FOR each marker in the text
|    SET markerPosition equal to the position of the marker in the text
|    CASE BASED on positiveFound AND negativeFound
|    |    CASE positiveFound is TRUE AND negativeFound is TRUE
|    |    |    SET deltaPositive equal to positivePosition minus markerPosition
|    |    |    SET deltaNegative equal to negativePosition minus markerPosition
|    |    |    IF textVerse is right THEN
|    |    |    |    IF deltaPositive is lesser than deltaNegative AND deltaPositive is greater than 0 THEN
|    |    |    |    |    SET markerQuality[i] equal to marker trailed with "+"
|    |    |    |    ELSE IF deltaNegative is lesser than deltaPositive AND deltaNegative is greater than 0 THEN
|    |    |    |    |    SET markerQuality[i] equal to marker trailed with "-"
|    |    |    |    ENDIF
|    |    |    ELSE
|    |    |    |    IF deltaPositive is lesser than delyaNegative AND deltaNegative is greater than 0 THEN
|    |    |    |    |    SET markerQuality[i] equal to marker trailed with "+"
|    |    |    |    ELSE IF deltaNegative is lesser than deltaPositive AND deltaPositive is greater than 0 THEN
|    |    |    |    |    SET markerQuality[i] equal to marker trailed with "-"
|    |    |    |    ELSE IF deltaPositive is lesser than deltaNegative AND deltaNegative is lesser than 0 THEN
|    |    |    |    |    SET markerQuality[i] equal to marker trailed with "-"
|    |    |    |    ELSE IF deltaNegative is lesser than deltaPositive AND deltaPositive is lesser than 0 THEN
|    |    |    |    |    SET markerQuality[i] equal to marker trailed with "+"
|    |    |    |    ENDIF
|    |    |    ENDIF
|    |    CASE positiveFound is TRUE AND negativeFound is FALSE
|    |    |    SET markerQuality[i] equal to marker trailed with "+"
|    |    CASE positiveFound is FALSE AND negativeFound is TRUE
|    |         SET markerQuality[i] equal to marker trailed with "-"
|    ENDCASE
|    SET i equal to i plus 1
ENDFOR
```

CASE B is:

```
SET i equal to 0
FOR each marker in the text
        SET tempMarkerQualityPlus equal to marker trailed with "+"
        SET tempMarkerQualityDash equal to marker trailed with "-"
        IF tempMarkerQualityPlus is in the text THEN
                SET markerQuality[i] equal to tempMarkerQualityPlus
        ELSE IF tempMarkerQualityDash is in the text THEN
                SET markerQuality[i] equal to tempMarkerQualityDash
        ENDIF
        SET i equal to i plus 1
ENDFOR
```

CASE C is:

```
SET i equal to 0
FOR each marker in the text
        SET markerFound equal to FALSE
        FOR each qualifier in positiveQualifiers
                SET tempMarkerQualityPlus equal to marker trailed with qualifier
                IF tempMarkerQualityPlus is in the text THEN
                        SET markerQuality[i] equal to tempMarkerQualityPlus
                        SET markerFound equal to TRUE
                ENDIF
        ENDFOR
        IF markerFound is FALSE THEN
                FOR each qualifier in negativeQualifiers
                        SET tempMarkerQualityDash equal to marker trailed with qualifier
                        IF tempMarkerQualityDash is in the text THEN
                                SET markerQuality[i] equal to tempMarkerQualityDash
                        ENDIF
                ENDFOR
        ENDIF
        SET i equal to i plus 1
ENDFOR
```

CASE D is:

```
SET i equal to 0
FOR each percentageValue in the text
    SET percValue[i] equal to percentageValue
    SET deltaPerc equal to 10^9
    SET percPosition equal to the percentageValue position in the text
    FOR each marker in text
        SET markerPosition equal to the position of the marker in the text
        SET deltaPos equal to percPosition minus markerPosition
        IF deltaPos is lesser than deltaPerc THEN
            SET markerPerc[i] equal to marker
        ENDIF
    ENDFOR
ENDFOR
```

Table S5. Data dictionary extracted from REDCap for data fields used to map each word detected from the NLP.

VARIABLE/FIELD NAME	DETECTABLE FROM THE NLP WEB APPLICATION?	REDCAP INSTRUMENT	FIELD TYPE	FIELD LABEL	CHOICES, CALCULATIONS, OR SLIDER LABELS	TEXT VALIDATION TYPE OR SHOW SLIDER NUMBER
hist_executed_req	Y	disease_parameters	yesno	Histopathological examination executed?		
diagnosis_dis	Y	disease_parameters	text	Diagnosis (semi-automatic)		
ln_specimen_dis	Y	disease_parameters	yesno	Did the specimen type a lymph-node?		
internal_ihc_req	Y	disease_parameters	yesno	Was the exam performed internally to the institution?		
external_exam_req	Y	disease_parameters	text	If external, please specify the centre name		
nod_exam_num_req	Y	disease_parameters	text	Number of the exam		
nod_date_exam_req	Y	disease_parameters	text	Date of the exam		date_dmy
protein_ihc_dis	Y	disease_parameters	yesno	Was the immunohistochemistry performed?		
blastoid_dis	Y	disease_parameters	yesno	Has a blastoid histology been detected?		
bcl2_ln_exp_dis	Y	disease_parameters	radio	BCL2 detected?	1, Yes | 0, No	
myc_ln_exp_dis	Y	disease_parameters	radio	MYC detected?	1, Yes | 0, No	
bcl6_ln_exp_dis	Y	disease_parameters	radio	BCL6 detected?	1, Yes | 0, No	
cd5_ln_exp_dis	Y	disease_parameters	radio	CD5 detected?	1, Yes | 0, No	
cd10_ln_exp_dis	Y	disease_parameters	radio	CD10 detected?	1, Yes | 0, No	
cd15_ln_exp_dis	Y	disease_parameters	radio	CD15 detected?	1, Yes | 0, No	
cd19_ln_exp_dis	Y	disease_parameters	radio	CD19 detected?	1, Yes | 0, No	
cd20_ln_exp_dis	Y	disease_parameters	radio	CD20 detected?	1, Yes | 0, No	
cd22_ln_exp_dis	Y	disease_parameters	radio	CD22 detected?	1, Yes | 0, No	
cd23_ln_exp_dis	Y	disease_parameters	radio	CD23 detected?	1, Yes | 0, No	
cd30_ln_exp_dis	Y	disease_parameters	radio	CD30 detected?	1, Yes | 0, No	
cd43_ln_exp_dis	Y	disease_parameters	radio	CD43 detected?	1, Yes | 0, No	
cd79a_ln_exp_dis	Y	disease_parameters	radio	CD79a detected?	1, Yes | 0, No	

cget_ln_exp_dis	Y	disease_parameters	radio	GCET detected?	1, Yes | 0, No	
gcet2_ln_exp_dis	Y	disease_parameters	radio	GCET2 (HGAL) detected?	1, Yes | 0, No	
foxp1_ln_exp_dis	Y	disease_parameters	radio	FOXP1 detected?	1, Yes | 0, No	
irf4_ln_exp_dis	Y	disease_parameters	radio	IRF4/MUM1 detected?	1, Yes | 0, No	
lmo_ln_exp_dis	Y	disease_parameters	radio	LMO2 detected?	1, Yes | 0, No	
igd_ln_exp_dis	Y	disease_parameters	radio	IGD detected	1, Yes | 0, No	
igm_ln_exp_dis	Y	disease_parameters	radio	IGM detected	1, Yes | 0, No	
pax5_ln_exp_dis	Y	disease_parameters	radio	PAX5 detected?	1, Yes | 0, No	
fmc7_ln_exp_dis	Y	disease_parameters	radio	FMC7 detected?	1, Yes | 0, No	
cd1_ln_exp_dis	Y	disease_parameters	radio	Cyclin D1	1, Yes | 0, No	
bcl2_positive_dis	Y	disease_parameters	text	BCL2 positive cells		integer
myc_positive_dis	Y	disease_parameters	text	MYC positive cells		integer
mum1_positive_dis	Y	disease_parameters	text	MUM1 positive cells		integer
ki67_positive_dis	Y	disease_parameters	text	Ki-67 positive cells		integer
coo_exe_hans_dis	Y	disease_parameters	yesno	Was the Subtype Classification executed according to Hans?		
coo_hans_dis	Y	disease_parameters	radio	Subtype classification according to Hans	1, GCB | 2, Not GCB	
fish_exe_dis	Y	disease_parameters	yesno	Was the FISH executed?		
internal_fish_req	Y	disease_parameters	yesno	Was the exam performed internally to the institution?		
external_fish_req	Y	disease_parameters	text	If external, please specify the centre name		
fish_date_exam_req	Y	disease_parameters	text	Date of the analysis		date_dmy
fish_exam_num_req	Y	disease_parameters	text	Number of the exam		
myc_rear_dis	Y	disease_parameters	radio	MYC rearrangement detected?	1, Yes | 0, No	
bcl2_rear_dis	Y	disease_parameters	radio	BCL2 rearrangement detected?	1, Yes | 0, No	
bcl6_rear_dis	Y	disease_parameters	radio	BCL6 rearrangement detected?	1, Yes | 0, No	
bm_dis	Y	disease_parameters	yesno	Is present a medullary disease?		
bm_specimen_dis	Y	disease_parameters	yesno	Was the bone marrow analysed by IHC?		
internal_ihc_bm_req	Y	disease_parameters	yesno	Was the exam performed internally to the institution?		

external_exam_bm_req	Y	disease_parameters	text	If external, please specify the centre name		
bm_exam_num_req	Y	disease_parameters	text	Number of the exam		
bm_date_exam_req	Y	disease_parameters	text	Date of the analysis		date_dmy
cd5_bm_exp_dis	Y	disease_parameters	radio	CD5 detected?	1, Yes | 0, No	
cd10_bm_exp_dis	Y	disease_parameters	radio	CD10 detected?	1, Yes | 0, No	
cd15_bm_exp_dis	Y	disease_parameters	radio	CD15 detected?	1, Yes | 0, No	
cd19_bm_exp_dis	Y	disease_parameters	radio	CD19 detected?	1, Yes | 0, No	
cd20_bm_exp_dis	Y	disease_parameters	radio	CD20 detected?	1, Yes | 0, No	
cd22_bm_exp_dis	Y	disease_parameters	radio	CD22 detected?	1, Yes | 0, No	
cd23_bm_exp_dis	Y	disease_parameters	radio	CD23 detected?	1, Yes | 0, No	
cd30_bm_exp_dis	Y	disease_parameters	radio	CD30 detected?	1, Yes | 0, No	
cd43_bm_exp_dis	Y	disease_parameters	radio	CD43 detected?	1, Yes | 0, No	
cd79a_bm_exp_dis	Y	disease_parameters	radio	CD79a detected?	1, Yes | 0, No	
myc_bm_exp	Y	disease_parameters	radio	MYC detected?	1, Yes | 0, No	
gcet_bm_exp_dis	Y	disease_parameters	radio	GCET detected?	1, Yes | 0, No	
gcet2_bm_exp_dis	Y	disease_parameters	radio	GCET2 (HGAL detected ?)	1, Yes | 0, No	
foxp1_bm_exp_dis	Y	disease_parameters	radio	FOXP1 detected?	1, Yes | 0, No	
irf4_bm_exp_dis	Y	disease_parameters	radio	IRF4/MUM1 detected?	1, Yes | 0, No	
lmo_bm_exp_dis	Y	disease_parameters	radio	LMO2 detected?	1, Yes | 0, No	
igd_bm_exp_dis	Y	disease_parameters	radio	IGD detected?	1, Yes | 0, No	
igm_bm_exp_dis	Y	disease_parameters	radio	IGM detected?	1, Yes | 0, No	
pax5_bm_exp_dis	Y	disease_parameters	radio	PAX5 detected?	1, Yes | 0, No	
fmc7_bm_exp_dis	Y	disease_parameters	radio	FMC7 detected?	1, Yes | 0, No	
cd1_bm_exp_dis	Y	disease_parameters	radio	Cyclin D1 detected?	1, Yes | 0, No	

Abbreviations. NLP, natural language process; Y, Yes; radio, radio button; CD, cluster of differentiation; IHC, immunohistochemical; FISH, fluorescent *in situ* hybridization.

Source code S1. Source code developed in Python for the application of the thesaurus rules.

```
#Thesaurus for the biomarkers
difficult_pattern = "IG.|BC.[0-9]|..67|CICLINAD.|MUM|LCA|CKAE1/AE3|CD31|P\d+\+|CD\d+|BC.E"
Used after the normalization:    text = text.replace(' ','')
   text = text.replace('\n','')
   text = text.replace('-','')

#Thesaurus for the specimens
pattern_nodal = "tumef|infono|odul|infonod|linfoadenopatia|laterocervical[e|i]|mediastinic[a|o]|linfonodoascellar[e|i]|ascella|linfonodolinguinale|…
alinfonodoinguinale|mediastinic."
   pattern_extra_nodal = "esticol|rene|tonsill|bronchiali|bronchi|polmonare|polmon.|tiroid|mammella|mammar|stomac|fegato|duoden|ileo|colon|…
lingua|pancrea|urinario|splenic|milza|renale|ileale|digiuno|gastric|parotide|ghiandol.|salivar.|rinofaringe|vescical.|vescica|cutaneo|cutanea|cerebellar.|…
epatic|celebral.|antro|retroperitoneal.|epatic.|gastric|extradurale|retto|rettale|linfoadenopati.|digiuno|ileale|palat|trachea"
   pattern_PB = "periferico|sangue"
   pattern_BOM="bom|steomidollar|idollar|spirat|gnab|trucut|osse.|cauda|sternale"
Questi per gli specimen

#Thesaurus for the disease nomenclature
| C82.9 Follicular non-Hodgkin's lymphoma, unspecified        | C82.9 LINFOMA FOLLICOLAR NON HODGKIN                          |
| C83.3 Diffuse non-Hodgkins lymphoma, large cell (diffuse) | C83.3 LINFOMA DIFFUSO NON HODGKIN, A GRANDI CELLULE (DIFFUSO) |
| C83.1 Diffuse non-Hodgkin's lymphoma, Small cleaved cell (diffuse) | C83.1 LINFOMA NON HODGKIN MANTELL |
```

References

1. Ruhl J, Adamo MP, Dickie L, Negoita S. Hematopoietic and Lymphoid Neoplasm Coding Manual, Bethesda, MD, US: 2020.
2. World Health Organization. Classification of diseases (ICD). [https://www.who.int/classifications/classification-of-diseases].
